# Mitochondrial Bioenergetics and Turnover during Chronic Muscle Disuse

**DOI:** 10.3390/ijms22105179

**Published:** 2021-05-13

**Authors:** Jonathan M. Memme, Mikhaela Slavin, Neushaw Moradi, David A. Hood

**Affiliations:** Muscle Health Research Centre, School of Kinesiology and Health Science, York University, Toronto, ON M3J 1P3, Canada; jmemme@yorku.ca (J.M.M.); mslavin@my.yorku.ca (M.S.); neushaw7@yorku.ca (N.M.)

**Keywords:** skeletal muscle atrophy, mitochondrial quality control, mitochondrial biogenesis, mitophagy, autophagy, apoptosis, muscle disuse, reactive oxygen species

## Abstract

Periods of muscle disuse promote marked mitochondrial alterations that contribute to the impaired metabolic health and degree of atrophy in the muscle. Thus, understanding the molecular underpinnings of muscle mitochondrial decline with prolonged inactivity is of considerable interest. There are translational applications to patients subjected to limb immobilization following injury, illness-induced bed rest, neuropathies, and even microgravity. Studies in these patients, as well as on various pre-clinical rodent models have elucidated the pathways involved in mitochondrial quality control, such as mitochondrial biogenesis, mitophagy, fission and fusion, and the corresponding mitochondrial derangements that underlie the muscle atrophy that ensues from inactivity. Defective organelles display altered respiratory function concurrent with increased accumulation of reactive oxygen species, which exacerbate myofiber atrophy via degradative pathways. The preservation of muscle quality and function is critical for maintaining mobility throughout the lifespan, and for the prevention of inactivity-related diseases. Exercise training is effective in preserving muscle mass by promoting favourable mitochondrial adaptations that offset the mitochondrial dysfunction, which contributes to the declines in muscle and whole-body metabolic health. This highlights the need for further investigation of the mechanisms in which mitochondria contribute to disuse-induced atrophy, as well as the specific molecular targets that can be exploited therapeutically.

## 1. Introduction

The adaptability of skeletal muscle to various external stimuli has profound ramifications for overall health. It is well-established that chronic exercise supports favourable adaptions in muscle that contribute to improved metabolic fitness, longevity and the absence of various diseases. In contrast, chronic muscle inactivity promotes diminished muscle quality along with muscle atrophy that is characterized by reductions in muscle fiber cross-sectional area (CSA), the net result of increased catabolism concomitant with reduced skeletal muscle protein synthesis [1,2,3,4,5,6]. Indeed, muscle atrophy is a prominent feature of numerous pathophysiological conditions, including metabolic diseases, cancers, AIDS, and respiratory diseases, among numerous others, and leads to further disease progression and reduced mobility with aging [7,8]. In the absence of disease, skeletal muscle disuse may be brought about by chronic sedentarism, periods of immobilization due to injury, bed rest as result of illness, or for a select few, exposure to microgravity. In these cases, muscle atrophy may occur in the affected limb or more broadly throughout the body, creating functional and metabolic derangements in the affected tissue. Given the frequent recruitment and activation of slow-twitch oxidative fibers for the maintenance of posture and other routine tasks, muscles with a predominantly type I fiber composition such as the soleus are more susceptible to chronic disuse than muscles with a more mixed fiber complexion, such as the gastrocnemius [9,10,11]. Prolonged inactivity promotes the acquisition of the structural, biochemical and mechanical properties of a glycolytic tissue within these type I fibers, and generates metabolic dysfunction emanating from the mitochondrial network. Thus, the effects of muscle atrophy with prolonged inactivity are variable and determined, in part, by the composition of fiber types within the affected tissue [9,12,13,14].

The World Health Organization posits that insufficient physical activity is the leading risk factor for the advancement of non-communicable diseases and diminished quality of life, and is a global phenomenon that requires a substantial and coordinated effort among nations to remediate the pervasive sedentarism [15]. The consequences of muscle atrophy are substantial, with implications in functional decline, disability, disease and premature death. Even during prescribed periods of muscle disuse, as part of therapeutic intervention following injury or illness, the resultant atrophy in the muscle can prove detrimental by delaying, impairing or even preventing adequate recovery, often despite rehabilitative intervention strategies [16,17,18,19,20]. As such, studies devoted to elucidating the molecular events underlying muscle atrophy and the accompanying metabolic impairments are of particular importance in identifying therapeutic targets and strategies to slow and/or halt muscle atrophy and metabolic decline in a variety of contexts. Numerous models of muscle disuse have been employed in both human and animal subjects, which allow researchers to investigate the specific etiology of muscle atrophy across a spectrum of physiological conditions.

## 2. Models of Muscle Disuse

### 2.1. Human Models of Muscle Disuse

Chronic inactivity in humans provides the most direct study of the conditions that bring about muscle wasting in patient populations. Unilateral limb immobilization/casting, as well as bed rest studies present the two most commonly used approaches for studying muscle wasting conditions in human subjects, and are particularly useful in studying muscle decline in the absence of associated comorbidities. Unilateral limb immobilization involves fixing a joint, such as the elbow or knee, in the flexed position while maintaining the limb suspended above the ground. This approach has been used for decades to compare the effects of muscle disuse in one limb, compared to the contralateral, unaffected limb [21]. The ability to localize muscle disuse with this model closely mimics the unloading that occurs following musculoskeletal injuries in the clinical setting, and allows for direct comparisons within the same subject, while eliciting pronounced reductions in CSA and muscle mass [22,23,24]. In fact, reports indicate that limb immobilization is capable of inducing 0.44% reduction in vastus lateralis mass per day [24,25,26]. Bed rest studies have also long been employed to simulate, not only prolonged periods of inactivity following injury or illness, but additionally, the muscle wasting conditions brought about by space flight [27]. Dry-immersion bed rest studies go one step further by positioning a subject in the supine position within a waterproof barrier and suspending them in thermoneutral water, in order to most accurately recreate the complete unloading that is experienced by astronauts in space [28]. The additional benefit of dry-immersion bed rest is the higher rate and greater extent at which neuromuscular adaptations are achieved, compared to traditional bed rest [28]. Both bed rest and limb immobilization studies allow effective countermeasures to be tested, in order to prevent, offset, or reverse the decline in muscle observed with disuse, while also providing a relevant model of the wasting associated with diseases and aging. In a clinical setting, brought into recent focus as a result of the COVID-19 pandemic, is the significant respiratory muscle atrophy and dysfunction resulting from mechanical ventilation in the intensive care unit. Respiratory muscle atrophy and dysfunction can occur in just 18 h following assisted ventilation [29,30]. Samples derived from these patients, although scarce, allow for direct study of the myopathy that makes these subjects unable to return to normal, unsupported ventilation [31,32].

### 2.2. Animal Models of Muscle Disuse

In many cases employing animal models of muscle inactivity offers greater flexibility and the ability to control for potential confounding variables, while also mimicking many of the relevant disuse atrophy-generating conditions that may affect humans [33,34]. In animals, hindlimb suspension and hindlimb immobilization are two common approaches for inducing disuse-atrophy, particularly in mice and rats. Hindlimb suspension involves affixing orthopedic tape to the tail of the animal and attaching the tape to a metal swivel located at the top of the cage, thus, allowing for unhindered 360° rotation and movement around the cage using the forelimbs [35,36,37,38]. The hindlimb suspension technique was first developed nearly 50 years ago by the National Aeronautics and Space Agency, in order to simulate weightlessness, and thus, makes the approach particularly useful for studying the effects of musculoskeletal unloading [39,40]. Similar to immobilization in humans, rodent hindlimb immobilization involves fixing one limb with a plastic brace, or within a plastic tube, in order to maintain the joint in a flexed position [36]. In this way, disuse can be accomplished by using, either a fixed dorsiflexion of the ankle joint to induce atrophy of the tibialis anterior and extensor digitorum longus, or fixed plantarflexion to produce atrophy of the gastrocnemius, plantaris, and soleus [41,42,43]. The benefits of employing hindlimb immobilization include the ability to compare the effects of disuse to the contralateral limb of the same animal, while restricting muscle contraction. Additionally, these techniques offer a relatively simple, cost effective approach to induce reductions in muscle CSA, mass, and strength in as little as one week [44]. Another model that may be applied to rodents involves confined housing via small cages that restrict movement, and thus, limit physical activity so as to replicate chronic sedentarism [45,46,47,48]. This model of restricted movement is useful in studies interested in observing the systemic effects of muscle inactivity such as the changes in glucose metabolism and insulin resistance, along with the muscle atrophy and myopathy that ensues [45,48,49]. Likewise, an added benefit to any of these aforementioned rodent models is the ability to study corrective interventions, such as re-training to minimize or to reverse the detrimental effects of muscle disuse.

In contrast to these relatively non-invasive techniques, denervation, or similarly, nerve crushing and tetrodotoxin (TTX) cuffing, provide surgical methods of inducing muscle disuse, and have applicability to severe spinal cord or neuronal injury, as well as aging [50]. Denervation involves the excision of a small (~2–3 mm) segment of the nerve innervating the target muscle. Generally the tibial nerve is targeted in rats, while the sciatic nerve is used in mice, thus, affecting the lower hindlimb muscles [36,51,52]. Denervation of the nerve completely abolishes nerve-muscle communication via neuromotor and neurotrophic inputs, leading to rapid atrophy of the tissue. Nerve crushing is similar to denervation, however it requires the application of a force to the nerve with adequate pressure to temporarily ablate neural input to the muscle, while still allowing for neural regeneration and re-innervation to occur over time [36,53,54]. Alternatively, treatment with the sodium channel blocking drug TTX provides a chemical approach to denervation. TTX cuffing around the nerve maintains axonal continuity to the muscle and vascular beds, along with the flow of trophic factors that are otherwise lost with mechanical denervation, yet eliminates the impulse conduction from the nerve to muscle in order to prevent muscle contraction [53,54]. Each of these approaches are both suitable and sufficient for inducing muscle disuse-induced atrophy as well as metabolic myopathy. However, the choice of which model is best depends on the context and application of the experiments, and any conclusions made using one technique should be carefully applied and contrasted with those obtained via other disuse methodologies or model organisms. 

## 3. Early Studies on Mitochondrial Changes with Disuse

The prevailing understanding of disuse-induced atrophy involves reductions in protein synthesis along with increased protein breakdown, oxidative stress, and inflammation leading to functional decline of the muscle [6,17,55]. Chronic muscle disuse results in the suppression of the IGF-Akt-mTOR axis, which is the predominant mediator of protein synthesis, and thus, muscle growth [6,26,56,57]. Muscle protein synthesis rates have been shown to be reduced as early as 6 h following the onset of muscle inactivity [4,34]. Meanwhile, the concurrent activation of proteolysis pathways further shift the balance in favour of muscle atrophy, with muscle protein breakdown believed to be the key contributor to this effect in animal models [6,58,59]. However, in humans, the impact of proteolysis in response to chronic muscle disuse is less conclusive [33,60,61,62]. Nevertheless, the primary proteolytic pathways mediating muscle atrophy include the autophagy/lysosome, as well as the ubiquitin-proteasome systems. The balance between protein synthesis and degradation is further maintained via the Akt-mediated inactivation of the transcription factors FoxO1 and FoxO3, which regulate the key ubiquitin ligases muscle ring-finger protein-1 (MuRF1) and muscle atrophy F-box (Atrogin-1) [50,63], along with a host of other genes involved in proteolysis [64,65,66]. Therefore, suppression of the IGF-Akt axis inherently favors the expression of the degradation machinery that promotes muscle atrophy.

The complexity in the regulation of muscle mass and function following periods of prolonged inactivity remains elusive, but increasing evidence suggests the important role mitochondria holds in mediating the declines in muscle mass and function following disuse conditions. Early research investigating muscle inactivity found a link between mitochondrial decline and muscle atrophy, and since these pioneering experiments, many other studies have reported concomitant alterations in mitochondrial morphology, volume, and function in inactive muscle [12,67,68]. Amid these early reports, it was determined that mitochondrial coupling was impaired 24 h following the onset of muscle disuse, suggesting that mitochondrial alterations are among the first changes to take place [69]. Mitochondrial enzyme activities, including cytochrome c oxidase and citrate synthase are reduced with muscle disuse as well, along with declines in ATP production rate. While this suggests the development of an energy deficiency [70], it should also be viewed in the context of the reduced energy demand brought about by muscle disuse. There is evidence to suggest that the reductions in mitochondrial function precede significant losses in muscle mass [71,72,73]. Studies in which mitochondrial function was artificially preserved or augmented during muscle disuse provide further insight into the role of mitochondria in determining changes in muscle mass during disuse. For instance, both transgenic overexpression of the master regulator of mitochondrial biogenies, peroxisome proliferator-activated receptor γ co-activator-1α (PGC-1α), or treatment with mitochondrial-targeted antioxidants, are sufficient to preserve muscle mass during hindlimb unloading [33,50,74,75]. Thus, a thorough understanding of the molecular events governing mitochondrial remodeling with chronic muscle disuse is of considerable interest in order to better understand the mechanisms of muscle atrophy, and to determine a potential avenue for therapeutic intervention.

## 4. Molecular Basis of Mitochondrial Decline during Disuse

**Figure 1:**
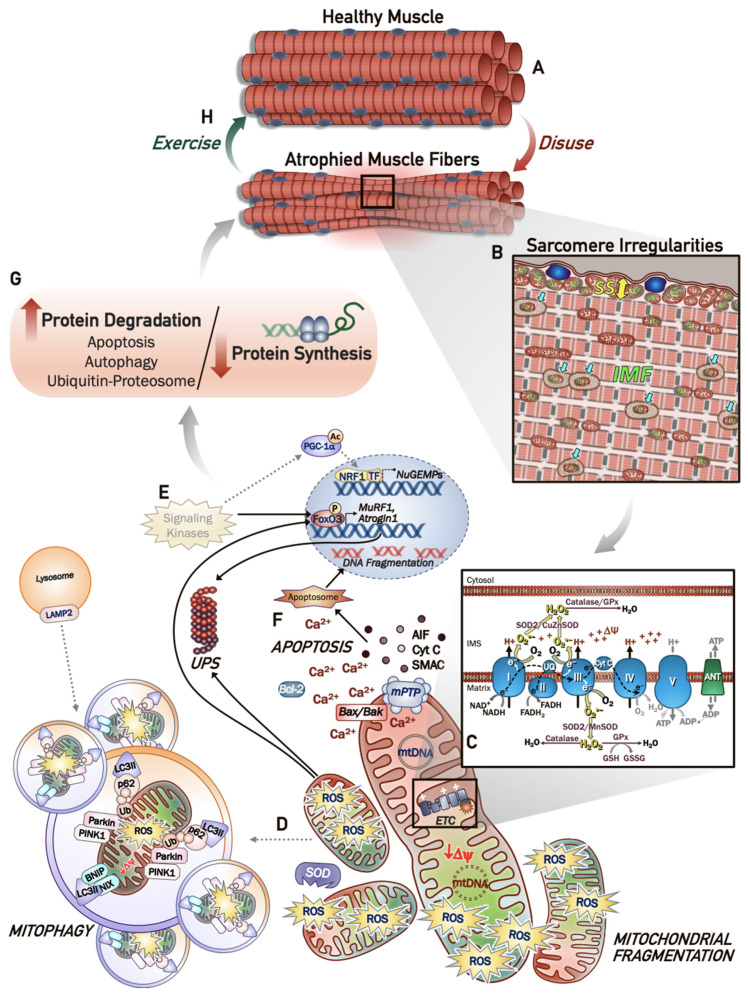
Disuse-induced mitochondrial derangements contribute to skeletal muscle fiber atrophy. (**A**) Atrophic regions of muscle fibers brought about following periods of prolonged muscle inactivity display marked alterations in mitochondrial morphology and function which can be visualized microscopically and have been determined experimentally. (**B**) Both subsarcolemmal (SS; yellow arrow) and intermyofibrillar (IMF) mitochondrial populations appear fragmented, with irregular shapes and unusual cristae formation indicative of dysfunctional organelles (green mitochondria). Defects in the mitochondrial turnover machinery contribute to the appearance of undegraded vacuoles, termed lipofuscin (blue arrows), which contribute to the irregularities within the sarcomere. (**C**) The absence of muscle contraction results in a decrease in the ADP supply, resulting in lower respiratory rates and an enhanced proton motive force (Δψ), thus promoting the creation of reactive oxygen species (ROS) in the form of superoxides (O_2_^•−^) and H_2_O_2_. Mitochondrial and cytosolic antioxidants neutralize superoxides, first via conversion to H_2_O_2_ by superoxide dismutases SOD1 and SOD2, then to water by antioxidant enzymes such as catalase or glutathione. As mitochondrial antioxidants are downregulated during muscle disuse, ROS accumulate in muscle mitochondria triggering the activation of degradation pathways. (**D**) Mitochondrial quality is maintained by the mitophagy machinery to selectively envelop dysfunctional organelles and delivers them to the lysosome for recycling. With chronic disuse, the process is impaired, leading to the accumulation of undegraded dysfunctional mitochondria. (**E**) In addition to this impaired turnover, activation of signaling kinases is reduced, leading to a diminished drive for mitochondrial biogenesis via PGC-1α, and promoting the expression of atrogenes, such as MuRF1 and atrogin1 that enhance protein degradation. (**F**) A consequence of the impaired mitochondrial quality control and chronic organelle dysfunction is the formation and opening of the mitochondrial permeability transition pore (mPTP), and subsequent release of pro-apoptotic factors such as AIF, Cytochrome c and SMAC, which lead to DNA fragmentation. (**G**) Collectively, these derangements contribute to enhanced protein degradation relative to protein synthesis, thus, exacerbating muscle atrophy. (**H**) Regular endurance exercise reverses many of the mitochondrial defects that are observed with chronic inactivity, and therefore is capable of preventing, diminishing or reversing disuse-induced muscle atrophy.

As mitochondria are malleable organelles, capable of calibrating both content and function to suit the cell’s energy demands, it is fitting that periods of inactivity lead to redundancy within the skeletal muscle mitochondrial network, thus contributing to compromised organellular function [76]. As mitochondrial derangements play a key role in the progression of disuse-induced muscle atrophy, it is important to understand the complexity of mitochondrial regulation and the processes that determine their content and function within muscle (Figure 1). Multiple pathways converge on the mitochondrial reticulum in order to regulate the stoichiometry between synthesis (mitochondrial biogenesis) and decomposition (mitophagy), and the dynamics of these processes have considerable influence on the concentration and quality of the organelle network in the muscle, which influence the metabolic health of the tissue.

### 4.1. Mitochondrial Biogenesis Signaling and the Effect of Muscle Disuse

Under healthy conditions, there are a series of events dedicated to the transcription, translation, import, and assembly of mitochondrial-specific complexes that maintain the organelle milieu [77]. The well-studied peroxisome proliferator-activated receptor γ co-activator-1α (PGC-1α) is an integral responder to physiological signals such as energetic stress as it is the key regulator of mitochondrial biogenesis. Under conditions of cellular stress, such as during exercise, PGC-1α may become activated by post-translational modifications such as phosphorylation [78], ubiquitination [79], or deacetylation by the NAD^+^-dependent protein deacetylase, sirtuin 1 (SirT1). This latter modification enhances PGC-1α’s role as a transcriptional co-activator of nuclear genes encoding mitochondrial proteins (NuGEMPs) [80,81,82]. Active PGC-1α partners with the transcription factors nuclear respiratory factor 1 and 2 (NRF-1 and 2), or estrogen-related receptor-α (ERR-α), among others to transcribe genes encoding mitochondrial-targeted proteins [83]. Newly made precursor proteins are unfolded and then cross the mitochondrial membranes by interacting with translocase of the outer membrane (TOM), translocase of the inner membrane (TIM), and pre-sequence translocase-associated motor (PAM) proteins, as they direct mitochondrial substrates to their appropriate sites, whereby they may be cleaved and refolded to serve as fully functional mitochondrial proteins [77]. Among the most notable matrix-destined proteins, is mitochondrial transcription factor A (Tfam), the most important regulator of mtDNA transcription [83]. However, this import process functions to translocate >1000 other proteins to appropriate locations within the organelle, serving as a critical step in organelle maintenance, expansion and biogenesis. Further growth of the mitochondrial network is accomplished through the recruitment of mitochondrial fusion proteins, dynamin-like 120 kDa protein (Opa1) and mitofusins 1&2 (Mfn1/2) and a concomitant reduction in fission proteins, dynamin related protein (Drp1) and mitochondrial fission 1 protein (Fis1), thus, creating a balance of morphology-regulating proteins that favours the formation of an organelle network, or reticulum [82,84]. The expansion of this network serves to shorten diffusion distances, as well as provide an efficient lipid bilayer transport system for diffusion of substrates and oxygen, a situation particularly relevant during conditions of increased energy demand.

Under conditions of chronic muscle disuse, however, the absence of a contractile activity stimulus not only downregulates mitochondrial biogenesis, but also compromises the utility and efficacy of the mitochondria already present within the reticulum (Figure 1) [85]. Disuse is a potent stressor that has a large impact on the quantity and quality of skeletal muscle mitochondria. Denervation, immobilization, hind-limb suspension and mechanical-assisted ventilation have reported prominent reductions in mitochondrial content within the inactive muscle [12,52,73,86,87,88,89,90]. Notably, the expression of PGC-1α is significantly impaired early-on during a disuse stimulus [90,91,92,93], coinciding with reductions in mitochondrial content and muscle mass [89,90,92]. With denervation, PGC-1α mRNA and protein content are reduced within as short a period as one day and can remain depressed over many weeks [90,94,95]. Similarly, hindlimb unloading or immobilization can produce marked decrements in PGC-1α mRNA and protein content [91,96]. Moreover, reductions in Tfam gene expression have been observed in unloaded tissue, while downregulations in the gene and protein expression of reliable mitochondrial markers, including those involved in oxidative phosphorylation, have been observed across many models of disuse [52,73,89,90,92,97,98,99]. In addition, chronic muscle disuse exerts a profound influence on mitochondrial morphology and dynamics by altering the fission/fusion regulatory protein balance. The levels of fission proteins, Drp1 and Fis1, remain elevated relative to fusion proteins, Opa1 and Mfn1, thus promoting a more fragmented mitochondrial network [86,88,97,98,100,101,102,103]. The consequence of this altered regulatory protein expression is evidenced by altered organelle morphology with disuse. Indeed, electron microscopy (EM) images show not only reduced mitochondria per area of muscle, but also highlight mitochondrial displacement within muscle fibres (Figure 1B). Following prolonged denervation, mitochondria appear to shift from their correct placement along the I-band, instead appearing near the A-band of the sarcomere [104]. Furthermore, inter-myofibrillar (IMF) mitochondria, following hindlimb suspension, appear swollen and irregular along with a >60% reduction in the subsarcolemmal (SS) mitochondrial layer lying proximal to the myonuclei [33,100]. Fragmentation of the mitochondrial network exacerbates organellular deficiencies by impairing protein import processes. These are required to integrate nuclear-encoded mitochondrial proteins into the newly formed organelle, thus, further attenuating mitochondrial biogenesis, while also promoting the accumulation of damaging reactive oxygen species (ROS; Figure 1D) [51,72,100,105]. While muscle inactivity promotes derangements in the mitochondrial network via impaired biogenesis signaling and morphology, deficiencies in the effective clearance of these damaged organelles, as described below, exacerbate the skeletal muscle mitochondrial dysfunction that is observed with chronic disuse.

### 4.2. Mitophagy: The Mitochondrial Autophagy-Lysosome Pathway

The process of mitophagy refers to a mitochondrial-specific form of autophagy, and is integral to the maintenance of mitochondrial quality in muscle [82,106]. Generally, mitochondrial fragmentation allows for the isolation of dysfunctional organelle fragments from the reticulum, and their subsequent PINK1/Parkin-mediated ubiquitination [107]. In healthy, unstressed mitochondria, PTEN-induced kinase 1 (PINK1) is imported into the mitochondria where it is cleaved by mitochondrial proteases mitochondrial intramembrane cleaving protease (PARL) and matrix metalloproteinase (MPP) [108,109]. The cleaved PINK1 then dissociates from the mitochondrion and is rapidly degraded by the ubiquitin proteosome system (UPS) [107,109]. Disruption of the mitochondrial membrane potential (∆Ψ) during organellular dysfunction promotes the stabilization of PINK1 on the outer mitochondrial membrane, leading to its homodimerization and autophosphorylation [108]. Phosphorylated PINK1 can then serve as an active kinase capable of phosphorylating and activating the E3 Ub ligase, Parkin, which in turn, poly-ubiquitinates target proteins on the outer membrane, such as voltage-dependent anion channel (VDAC) and Mfn2, thus, flagging the organelle to be engulfed by the phagophore [107,108]. A series of conjugation reactions mediated by a host of autophagy (ATG) proteins promotes the maturation of the double membrane phagophore structure, along with lipidation of the precursor microtubule-associated protein 1A/1B-light chain 3 (LC3)-I into LC3-II, allowing for it to become embedded in the vesicle [82,110]. The adaptor protein, sequestosome 1 (p62), plays an important role in the recognition and encapsulation of dysfunctional mitochondria by anchoring ubiquitin-tagged organelles to phagophore-bound LC3-II, forming the autophagosome (Figure 1D) [82,111,112]. Fully-formed autophagosomes then traverse microtubule tracks to the lysosome where they undergo fusion for organelle degradation.

The product of autophagosome-lysosomal fusion is termed the autophagolysosome. This occurs by way of Rab and Arf small GTPase proteins that, when membrane-bound with GTP, are active and able to recognize effector proteins such as SNAREs, SM (Sec1/Munc-18), and coat proteins to facilitate fusion [110,113]. Lysosomes contain a complex array of hydrolytic enzymes including proteases, lipases, and nucleases, which enable degradation of whole organelles into their constituents [114]. The regulation of lysosomal acidification (pH~4.5) is accomplished by the vacuolar ATPase (v-ATPase), a large multimeric channel that transports protons into the lysosomal lumen in an ATP-dependent manner [114,115,116]. Moreover, lysosomal biogenesis and autophagy proteins are regulated transcriptionally by transcription factor EB (TFEB), which relies on calcium signaling via the transient receptor potential cation channel, mucolipin-1 (MCOLN1), in order to mediate the dephosphorylation and subsequent nuclear translocation of TFEB [116,117].

The mitophagic clearance of malfunctioning organelles is a critical component of muscle health during chronic inactivity [118]. However, derangements in the regulation of mitochondrial clearance can also contribute to the accumulation of damaged, dysfunctional organelles within muscle [82]. Denervation-induced alterations in the mitophagy pathway within rodent skeletal muscle include increased expression of whole muscle and mitochondrial-localized, p62, LC3-II, and Parkin, while hindlimb unloading similarly resulted in increases in the mRNA and protein content of mitophagy markers [91,119]. Denervation-induced increases in the mitophagy machinery were observed concomitant with reductions in mitochondrial content measured by COX activity, suggesting enhanced drive for mitochondrial clearance, and are morphologically supported by marked reductions in the SS mitochondrial layer via EM [119]. Further morphological evidence reveals disruptions in mitochondrial cristae, a characteristic of suboptimal mitochondria, as well as the appearance of lipofuscin vesicles, which is indicative of insufficient cargo clearance by the lysosome (Figure 1B) [119]. However, the effects of muscle disuse on mitophagy flux and the efficiency of the autophagy and/or lysosomal machinery in facilitating the removal of harmful organelles, remains to be determined. Thus, the question remains whether the increase in mitophagy markers detected with chronic disuse actually aid in mitochondrial clearance, or the bi-product of impaired turnover. Early indications suggest that these processes are limited at the terminal stages of the pathway, where autophagosome clearance does not efficiently match the burden of the accumulation of damaged mitochondria [84,119,120,121,122]. It may be that mitophagy has a limited capacity to maintain mitochondrial quality under excessive cellular stress [123]. If mitochondrial content and function cannot be maintained during periods of muscle disuse, then, the resultant dysfunction of the organelle reticulum will continue to contribute to the muscle wasting that is characteristic of these conditions. For instance, denervation of PGC-1α knockout animals results in higher p62 expression compared to levels observed in wild-type animals, while PGC-1α overexpressing animals display attenuated increases in p62 [52]. This highlights the important integration between mitochondrial biogenesis and clearance for the maintenance of the reticulum, and suggests a role for PGC-1α in potentially mediating both aspects of mitochondrial turnover.

## 5. Functional Consequences of Muscle Disuse

### 5.1. Mitochondrial Respiration

Mitochondrial bioenergetics encompasses the cascade of metabolic reactions that occur to convert chemical energy stored within macronutrients into ATP. Oxidative phosphorylation involves the synthesis of ATP that is “coupled” with substrate oxidation through the electron transport chain located within the inner mitochondrial membrane. A series of enzymatic reactions in Krebs’ Cycle produce electron carriers, NADH and FADH_2_, which release their electrons upon oxidation by complexes I, and II in the respiratory chain, respectively, and provide the free energy that is necessary to pump hydrogen ions into the intermembrane space (IMS). The resultant electrochemical gradient represents a proton-motive force that serves as the potential energy to produce ATP from ADP and inorganic phosphate via Complex V, the ATP Synthase. The supply of ADP, generated by hydrolysis of ATP by myosin ATPases during contractile activity, is the rate-limiting step of ATP synthesis. Thus, during the absence of contractile activity brought about by muscle disuse, the reduced availability of ADP slows electron transport, enhancing the proton motive force and increases ROS production, leading to mitochondrial dysfunction (Figure 1C).

Under conditions of muscle disuse, the assessment of mitochondrial respiration and ROS production in permeabilized muscle fibres and isolated mitochondria has proven to be valuable in the characterization of the skeletal muscle physiological response. Disuse significantly affects the bioenergetic function of mitochondria, demonstrated by deficits in oxygen consumption across multiple models of disuse in both animals and humans [33,73,98,101,124]. Three days of hindlimb unloading in rodents and one week of bed rest in humans reduces the oxygen flux of the inactive muscle by approximately 30% [73,125,126]. Rodents subjected to denervation surgery begin to exhibit both morphological and functional impairments by day three post-surgery [51,105]. Reductions in muscle CSA occur concomitant with ~45% declines in respiration rate, as well as a four-fold increases in ROS in the inactive muscle.

Electron microscopy reveals the existence of discrete, yet interconnected populations of mitochondria within skeletal muscle. When studied in isolation, these mitochondrial fractions retain differing bioenergetic and adaptive characteristics in response to disuse. Although IMF mitochondria elicit greater absolute rates of respiration, SS mitochondria are more malleable, displaying ~45% reductions in oxygen consumption as compared to ~16% declines observed in the IMF fraction three days post-denervation. In fact, mitochondrial deficits are evident after just 12 h of machine-supported ventilation, evidenced by declines in respiration along with reductions in the respiratory control ratio (RCR), which signifies uncoupling of ATP production from electron flow [103,127,128]. These data indicate that mitochondrial dysfunction is an early event brought about by muscle inactivity. Moreover, the impaired mitochondrial function observed with muscle disuse coincides with ~30–50% decreases in the gene and protein expression of the fusion proteins Mfn2 and OPA1 [103,125], along with a 50% increase in the fission protein Drp1 [103]. The resulting increase in fragmentation of the organelle reticulum contributes to the dysfunction of the mitochondrial network by impairing the sharing of substrates to fuel electron transport, and thereby reducing ATP production [125,129]. Mitochondrial energetic stress resulting from enhanced fission and a breakdown of the reticular structure generates a reduced ATP:AMP ratio in the cell along with increases in ROS, contributing to the activation of the FoxO3 transcription factor through the upstream kinase AMPK [130]. The nuclear translocation of FoxO3 increases the expression of ubiquitin ligases, Atrogin-1 and MuRF1 (Figure 1E) [72,103,128,131]. Inactivity also causes a reduction in the phosphorylation of Akt and ribosomal S6 protein, indicating attenuated protein synthesis [72,73,89]. Collectively, these findings illustrate a catabolic cellular environment within muscle during disuse that is potentially driven, in part, by impairments in mitochondrial energetics [132]. In addition, the mitochondrial dysfunction-induced increase in the production of ROS exacerbates the activation of these proteolytic pathways, contributing to muscle atrophy during disuse conditions, as described below (Figure 1G).

### 5.2. Mitochondrial ROS Production and Ca^2+^ Homeostais

Mitochondrial ROS serve as important signaling molecules in the homeostatic regulation of cellular processes within muscle. Under normal conditions, skeletal muscle is able to manage ROS levels through endogenous antioxidant enzymes, such as superoxide dismutase (SOD), glutathione peroxidase (GPx) and catalase, as well as a host of other enzyme and non-enzymatic antioxidants, including thioredoxins, and glutathione, respectively [133]. However, the greater production of ROS, as observed during muscle disuse, appears to overwhelm antioxidant defenses, resulting in oxidative damage to the tissue, which promotes dysfunction and atrophy (Figure 1C). For example, ROS have been shown to accumulate by 4–7-fold in tibialis anterior muscle over the course of 3–21 days following denervation, leading to a reduction in muscle mass by 21 days [90]. Similar reductions in CSA and muscle weight have been observed after mechanical ventilation [103,128], hindlimb unloading [72,73] and denervation [51], with corresponding reductions in force production [103]. With chronic muscle disuse, release of H_2_O_2_ stimulates AMPK-mediated proteolytic pathways, including the ubiquitin-proteosome and autophagy-lysosome systems, leading to increased muscle protein degradation and fiber atrophy [99,103,128,130]. Similarly, one week of bed rest was shown to induce ~15% increases in H_2_O_2,_ which parallels the increases observed in rodent muscle following seven days of hindlimb suspension or immobilization [72,73]. Likewise, just 12 h of machine-assisted ventilation promotes increases in H_2_O_2_ along with 4-hydroxynonenal (4-HNE), an indicator of oxidative damage [103,127].

Chronically inactive muscles display concomitant decreases in the expression of the essential antioxidants that are required to mitigate the harmful effects of accumulated ROS [90,101,127,128,134,135]. The enhanced oxidative stress that is observed in diaphragm muscle during mechanical ventilation coincides with reductions in the activities of glutathione reductase and SOD2. Therefore, this underscores the contribution of disuse-induced impairments in antioxidant enzyme activity to the pathology of ventilator-induced diaphragmatic dysfunction (VIDD) [128]. The influential role of mitochondrial ROS production in triggering the development of atrophy during disuse is further evidenced by reductions in 4-HNE and caspase/calpain activity in animals treated with the organelle-targeted antioxidant, SS31 [134]. Moreover, the CSA of muscle fibres in both the soleus and plantaris were preserved upon treatment with SS-31. Following more prolonged muscle disuse, such as 14 days of hindlimb immobilization, affected muscles exhibit further increases in H_2_O_2_ of ~50–60%, accompanied by 2-fold increases in calpain and caspase activity, indicating enhanced proteolysis [134]. Chronic muscle disuse promotes a marked induction of Atrogin-1 and MuRF-1 mRNA [136], as well as increased LC3-II/I ratio and PINK1 protein expression, demonstrating activation of both the autophagy-lysosome and ubiquitin proteosome proteolytic systems [73,103,128]. Moreover, oxidative stress can also attenuate protein synthesis by reducing the phosphorylation of the 4E binding protein 1, preventing ribosomal assembly and mRNA translation initiation, thereby further promoting muscle atrophy [137,138,139].

Another way in which mitochondrial ROS have been implicated in disuse-induced atrophy is via oxidation of the ryanodine receptor (RyR1) on the sarcoplasmic reticulum (SR) leading to both mitochondrial and cytosolic Ca^2+^ overload. Mitochondria, through their direct interaction with the sarcoplasmic reticulum (SR) at mitochondrial associated membrane (MAM) contact sites, play a role in regulating skeletal muscle intracellular Ca^2+^ levels by taking up calcium directly into the organelle [140]. While, Ca^2+^ passes through the OM via the voltage-dependent anion channel (VDAC), the IM is largely impermeable to ions, thus Ca^2+^ intake to the matrix is regulated by the mitochondrial calcium uniporter (MCU), which relies on the relatively negative membrane potential of the IM for Ca^2+^ entry [140,141,142]. Indeed, moderate influx of Ca^2+^ into the organelle is important in facilitating oxidative phosphorylation in muscle mitochondria [143,144,145]. However, the increased levels of mitochondrial ROS that occur with chronic disuse lead to the oxidation of the RyR1, which promotes excessive Ca^2+^ leak into the cytosol as well as an overload of Ca^2+^ that is taken up by mitochondria [146]. As a result, the elevated levels of Ca^2+^ lead to mitochondrial swelling and subsequent release of pro-apoptotic factors, as well as the activation of various proteolytic and apoptotic factors within the cytosol that collectively promote muscle atrophy via DNA fragmentation and myonuclear decay [33].

### 5.3. Mitochondrially-Mediated Apoptosis and Fibre Atrophy

As described above, mitochondrial dysfunction can also contribute to disuse muscle atrophy through the release of pro-apoptotic factors, which triggers a series of molecular events leading to myonuclear decay, termed apoptosis (Figure 1F) [147]. While different apoptotic cascades exist, the intrinsic mitochondrial apoptotic pathway initiates a series of molecular signals that contribute to DNA fragmentation and localized muscle atrophy [148,149]. In response to ROS and elevation in intracellular Ca^2+^, the mitochondrial permeability transition pore (mPTP) opens, which causes an equilibration of solutes and the influx of water into the mitochondrion, thereby, leading to organelle swelling and the subsequent release of pro-apoptotic factors [150]. Bcl-2 associated protein X (BAX) and Bcl-2 homologous antagonist/killer (BAK) assist with mitochondrial permeabilization in response to chronic cellular stress. While the mechanisms in which BAX/BAK promote mitochondrial membrane permeabilization remain incompletely understood, cyclin C has been implicated as a key protein involved in initiating BAX recruitment to the mitochondrion, specifically when the organelle is undergoing fission [151]. The subsequent oligomerization of Bax contributes to mitochondrial permeabilization and the release of cytochrome c (cyt-c) and other pro-apoptotic factors such as apoptosis inducing factor (AIF), as well as the small mitochondria-derived activator of caspase (SMAC) [152,153]. SMAC binds to and deactivates inhibitor of apoptosis (IAP), while cyt-c binding to repeat domains on adapter protein apoptotic protease activating factor 1 (APAF1) promotes the assembly of the apoptosome, which initiates a series of cascading caspase activation events [154,155]. Pro-caspase 9, upon recruitment to the APAF1-apoptosome complex, is cleaved to its mature form, caspase 9 [156]. Caspase 9 can then activate the effector caspases 3 and 7, which in turn, promote nuclear decay via nuclease-mediated DNA fragmentation (Figure 1F) [147,156,157].

Various models of muscle disuse display increases in apoptosis, inducing factor (AIF) and pro-apoptotic proteins BAX/BAK, which outweigh the expression of the anti-apoptotic protein Bcl-2 [86,90,101,158,159,160,161]. Indeed, five days of denervation resulted in a five-fold increase in the BAX/Bcl-2 ratio, which further increased to 10-fold following 21 days of denervation, suggesting an enhanced apoptotic drive as prolonged muscle inactivity ensues [90]. BAX/BAK double knockout mice showed attenuated apoptotic signaling in the absence of these proteins, and a 40% attenuation of fibre atrophy after seven days of denervation, revealing the importance of these proteins in the disuse-induced activation of apoptosis leading to muscle atrophy [124]. Apoptotic signaling is associated with alterations in the pore opening kinetics of the mPTP. Chronic muscle disuse elicited a 20% faster rate of mPTP opening, causing augmented cytochrome c release and an increase in apoptotic nuclei [90,159]. The development of muscle atrophy via nuclear decay mediated by this pathway is illustrated by serial histochemical sections, showing fiber “disappearance” in regions of the muscle where apoptotic signaling has occurred, marked by increases in caspase-3 and the degradation of nuclei [162]. Subsequently, local atrophy is revealed by reductions in fibre size and contractile force, revealing a physiological role for the link between mitochondrial dysfunction and apoptosis in contributing to atrophy of skeletal muscle [51,72,73,103,159].

## 6. Exercise Induces Changes in Cellular Signaling Pathways

The physiological profile of chronically disused skeletal muscle includes atrophy and weakness due to reduced protein synthesis and poor mitochondrial function [163]. Exercise has logically been suggested to have therapeutic potential, an area of research that remains to be of considerable interest. Increased physical activity has often been demonstrated to exert rescuing effects on disused skeletal muscle as a result of its ability to upregulate all aspects of mitochondrial quality control [82,164]. Both exercise preconditioning and reconditioning can reverse the atrophic muscular profile developed during a period of inactivity, through (1) enhancement of mitochondrial content and respiration, (2) attenuation of ROS production, and (3) reduced signaling within apoptotic and proteolytic pathways. Thus, exercise serves to protect, or reverse the structural and oxidative damage that accompanies disuse in skeletal muscle, while also improving protein synthesis rates, as well as the clearance of suboptimal mitochondria (Figure 1H) [93,99,102,127,165,166,167,168,169,170,171].

### 6.1. Exercise Restores the Mitochondrial Network

Exercise increases regulators of mitochondrial biogenesis, and improves fission:fusion characteristics to favour either the preservation or restoration of the mitochondrial reticulum following disuse. Contractile activity, in the form of preconditioning or reloading has been shown to improve the deficits in mitochondrial content, observed during both mechanical ventilation and hindlimb unloading. For example, exercise training improved PGC-1a mRNA levels 2–4-fold in comparison to muscle that did not undergo exercise preconditioning [127,172]. Rats allowed one-week of remobilization (cast removal), following seven days of unilateral hindlimb immobilization, displayed restored markers of mitochondrial biogenesis including PGC-1α, NRF-1, and Tfam, thus, promoting an increase in mitochondrial content [84]. Additionally, increased Parkin and LC3-II protein expression were reported with remobilization, suggesting improved mitophagy efficiency. Altogether, remobilization prompted a restoration in energy demand of the tissue, and re-established the maintenance of mitochondrial quality control [84]. Another study subjected aged male Wistar rats to one week of immobilization followed by an aerobic or resistance training intervention [173]. The CSA of the plantaris muscle with aerobic training was significantly improved compared to either the resistance re-training or no retraining cohorts, along with reduced ubiquitin-proteasome activity, and an upregulation in PGC-1α expression [173].

While, rescue effects can be seen with exercise following disuse, prior exercise may also prevent the accelerated protein degradation that occurs with disuse, as pre-training promotes mitochondrial adaptations that prevent mtDNA damage and ROS accumulation following subsequent periods of disuse [174]. Exercise performed over two weeks prior to seven days of hindlimb unloading increased the gene and protein expression of PGC-1α to levels similar to those observed in control animals, with a three-fold increase in Tfam mRNA and augmented COX-I protein expression, a product of mtDNA [93]. A regimen of chronic contractile activity imposed prior to seven days of denervation improved COX activity by 40%, augmenting mitochondrial content that preserved mitochondrial volume after a subsequent period of denervation [101]. Another study subjected mice to two-weeks of endurance training prior to a seven-day hindlimb suspension protocol and observed lower oxidative stress, measured by DHE staining, as well as a concomitant increase in antioxidant levels of mitochondrial SOD-1 and SOD-2 gene expression, as compared to hindlimb suspension alone. The increased antioxidant capacity and reductions in free radicals appear to protect mtDNA, thus leading to fewer mtDNA mutations, and coincided with the preservation in the expression of Tfam, citrate synthase, and COX-I [93]. Additional results from the same study showed that pre-training prior to hindlimb suspension retained the CSA of the soleus muscle as it remained similar to control levels. This suggests that pre-training prevents the accelerated reduction in CSA evident with disuse [93]. To date, there have been no reports on the effects of pre-training on markers of mitophagy or on lysosomal regulation. While exercise following disuse is a well-established solution to rescuing mitochondrial content by way of augmented mitochondrial quality control, future studies aimed at characterizing the threshold for mitochondrial preservation with pre-exercise models are warranted. These studies could help in establishing the potential therapeutic effects of exercise in mitigating muscle decline in the context of various disease states, or periods of microgravity. 

### 6.2. The Rescuing Effects of Exercise on Mitochondrial Function

The restoration of mitochondrial biogenesis signaling with exercise following muscle disuse is accompanied by the recovery of mitochondrial energetics. This is revealed by an increased RCR by 30% in the diaphragms of exercised animals that had previously undergone mechanical ventilation, representing a substantial improvement in the disuse-induced reduction in the RCR, and protection against mitochondrial uncoupling [127,128] The decline in mitochondrial energetics and content that occurs during disuse can also be reversed with reinstated ambulation after periods of inactivity. Following 10 days of hindlimb suspension, muscle that is reloaded for three days regained mitochondrial mass evidenced by an upregulation in cardiolipin content, an inner mitochondrial phospholipid responsible for maintaining mitochondrial cristae formation, and electron transport chain function [72]. The amelioration of mitochondrial content with reloading is also associated with a two-fold improvement in endurance capacity [99]. Seven days of reloading elicited improved mitochondrial oxygen consumption by 40%, with a further increase of 10% by 21 days, and an enhancement in mitochondrial gene expression leading to a complete rescuing of muscle weight [73]. In conjunction with an expanded mitochondrial pool, the improvement in mitochondrial bioenergetics with exercise reloading creates a more aerobic phenotype and contributes to a healthier muscle profile following periods of disuse.

As described above, the source of enhanced mitochondrial ROS during muscle disuse is due, in part, to decreased levels of antioxidants, along with the low rate of mitochondrial respiration, as a result of the low ATP demand, causing electrons to leak from the ETC to form free radicals [175]. These detrimental changes can be reversed with exercise, as exercise increases antioxidant protein expression, while also promoting an increase in the hydrolysis of ATP into ADP, thereby helping to reduce ROS emissions. Exercise preconditioning over a period of 10 days prior to 12 h of mechanical ventilation attenuated H_2_O_2_ release in isolated diaphragmatic mitochondria, as well as 4-HNE production, an indicator of lipid peroxidation to levels similar to control animals [127,128]. The mitochondrial antioxidant enzymes SOD1, SOD2, GPx and Catalase, were upregulated by 40% in the preconditioned animals compared to the non-exercised group, measured in both isolated mitochondria and whole muscle homogenates [127]. This enhanced antioxidant capacity most likely contributes to the decrease in H_2_O_2_ emission during exercise. In the muscle of animals subjected to exercise preconditioning for two weeks before hindlimb suspension, a three-fold upregulation in SOD2 expression and a 40% increase in protein levels was observed [93]. Interestingly, ROS production in IMF mitochondria was attenuated in muscle that was electrically stimulated to contract prior to denervation, and the muscle retained higher MnSOD protein expression compared to non-stimulated muscle [101]. Following seven days of hindlimb unloading, hindlimb reloading promoted an 80% reduction in malondialdehyde (MDA) contents, a cellular product of damage to lipids and DNA [99].

The upregulation in ROS emission in sedentary muscle is an influential effector in the activation of proteolytic pathways, muscle atrophy and a loss of contractile strength. The development of atrophy and subsequent dysfunction occurs via increased signaling within apoptotic pathways and the UPS pathway, as discussed above. However, chronic exercise lowers the expression of major signaling molecules in these pathways, attenuating the apoptotic and proteolytic vulnerability of muscle cells during disuse. In exercise-preconditioned diaphragms that are exposed to mechanical ventilation, a 30–40% reduction in the protein expression of Calpain 1 and caspase-3, paired with 70–80% reductions in mRNA levels of Atrogin-1 and MuRF-1 was observed [127]. This evidence is coupled with improvements in diaphragmatic force generation and the rescuing of the CSA of all fibre types, indicating attenuated atrophy [127]. Exercise preconditioning reduced MuRF-1 and Atrogin-1 mRNA by 50–70% during mechanical ventilation, effects that were abolished upon the treatment of exercised animals with an antisense oligonucleotide targeted against SOD2 [128]. The reduced expression of SOD2 additionally prevented the exercise-induced rescuing of the CSA of diaphragm fibers subjected to mechanical ventilation, suggesting a causal role of ROS production in promoting muscle atrophy via the UPS pathway [128]. Muscle reloading following hindlimb suspension results in improved expression of upstream signaling kinases regulating muscle protein turnover, including the increased expression of phosphorylated Akt and its target, phosphorylated Foxo3, preventing its nuclear localization and subsequent transcription of its downstream targets, including atrogenes and caspases [72]. Akt additionally stimulates anabolism and protein synthesis by phosphorylating mTOR, which in turn, phosphorylates ribosomal protein S6 to activate translational initiation [73]. These alterations promote ribosomal biogenesis and myofibrillar protein synthesis, while reducing proteolysis, thus, contributing in the reversal of muscle atrophy induced by hindlimb unloading [72,176].

## 7. Conclusions

Muscle disuse initiates signaling pathways that, over time, lead to muscle atrophy and weakness. Periods of inactivity, even as a prescribed rehabilitative program following severe injury or illness, can generate mitochondrial derangements that promote secondary consequences, including reductions in skeletal muscle mass, strength and endurance. The resultant muscle decline presents an increased risk of further debilitation and the advancement of various metabolic and cardiorespiratory diseases. Mitochondria play a role in the regulation of muscle mass and quality via retrograde signaling to the nucleus involving ROS, energy deficits and apoptosis. Therefore, maintenance of the organelle reticulum is critical to either offset or minimize the detrimental outcomes of chronic disuse. The therapeutic effects of exercise have been observed to mitigate and/or reverse the disuse-induced maladaptive phenotype by restoring the signaling events that initiate mitochondrial biogenesis and proper organelle clearance, while also abolishing the harmful levels ROS via restoration of mitochondrial antioxidants. The evidence reveals that sustained muscle contractions, as with aerobic exercise, greatly improve the mitochondrial derangements that develop during periods of inactivity, with considerable research highlighting the utility of both exercise preconditioning and reconditioning/reloading. Preconditioning is particularly effective in preventing mitochondrial decay to suboptimal levels following periods of disuse. Rehabilitative training following periods of inactivity is sufficient to re-establish mitochondrial volume and function within the muscle, thus, restoring muscle mass, strength and endurance back toward normal levels. Thus, the beneficial outcomes of contractile activity provide optimism in improving muscle health and the quality of life of those subjected to extended periods of inactivity. Future work in this area should include further delineation of the mechanisms connecting disuse-induced mitochondrial dysfunction and the expression of genes controlling muscle wasting, and specifically which potential molecular targets can be exploited to regulate mitochondrial health in the face of atrophic conditions.

## Author Contributions

All authors contributed to the writing of this manuscript. J.M.M. and D.A.H. were responsible for conceptualizing and editing of the review. All authors have read and agreed to the published version of the manuscript.

## Data Availability

Not applicable.

## Abbreviations

**ΔΨ**–mitochondrial membrane potential; **4-HNE**–4-Hydroxynonenal; **ADP**–adenosine diphosphate; **AIF**–apoptosis inducing factor; **Akt**–protein kinase B; **AMP**–adenosine monophosphate; **AMPK**–AMP-activated kinases; **APAF1**–Apoptotic protease activating factor 1; **ATP**–adenosine triphosphate; **ATG**; autophagy related protein; **Atrogin1**–muscle atrophy F-box (MAFbx); **BAK**–Bcl-2 homologous antagonist/killer; **BAX**–Bcl-2-associated X protein; **Bcl-2**–B-cell lymphoma 2; **COX**–cytochrome c oxidase; **CSA**–cross-sectional area; **Drp1**–dynamin-related protein-1; **ERRα**–estrogen related receptor α; **ETC**–electron transport chain; **FADH_2_**–flavin adenine dinucleotide + hydrogen; **Fis1**–mitochondrial fission 1 protein; **FoxO1 & 3**–forkhead box protein O-1 & 3; **GPx**–glutathione peroxidase; **GTP**; guanosine triphosphate; **IAP**–inhibitor of apoptosis; **IGF**–insulin-like growth factor; **IMF**–intermyofibrillar mitochondria; **IMS**–intermembrane space; **LC3 I & II**–microtubule-associated proteins 1A/1B light chain 3B; **MCOLN1**–mucolipin-1; **Mfn1**–mitofusin-1; **MPP**–matrix metalloproteinases; **mPTP**–mitochondrial permeability transition pore; **mTOR**–mechanistic target of rapamycin; **MuRF1**–muscle ring-finger protein-1; **NADH**–nicotinamide adenine dinucleotide + hydrogen; **NRF1 & 2**–nuclear respiratory factor-1 & 2; **NuGEMPs**–nuclear genes encoding mitochondrial proteins; **Opa1**–dynamin-like 120 kDa protein; **p62**–sequestosome 1; **PAM**–presequence translocase-associated motor protein; **PARL**–mitochondrial intramembrane cleaving protease; **PGC-1α**–peroxisome proliferator-activated receptor γ coactivator-1α; **PINK1**–phosphatase and tensin homolog (PTEN)-induced kinase 1; **RCR**–respiratory control ratio; **ROS**–reactive oxygen species; **SirT1**–sirtuin1; **SMAC**–small mitochondria-derived activator of caspase; **SNARE**–Soluble NSF attachment protein receptor; **SOD**–superoxide dismutase; **SS**–subsarcolemmal mitochondria; **Tfam**–mitochondrial transcription factor A; **TFEB**–transcription factor EB; **TIM**–translocase of the inner membrane; **TOM**–translocase of the outer membrane; **TTX**–tetrodotoxin; **v-ATPase**–vacuolar ATPase; **VDAC**–voltage dependent anion channel.
